# Supramolecular Packing Drives Morphological Transitions of Charged Surfactant Micelles

**DOI:** 10.1002/anie.202004522

**Published:** 2020-08-17

**Authors:** Ken Schäfer, Hima Bindu Kolli, Mikkel Killingmoe Christensen, Sigbjørn Løland Bore, Gregor Diezemann, Jürgen Gauss, Giuseppe Milano, Reidar Lund, Michele Cascella

**Affiliations:** ^1^ Department Chemie Johannes Gutenberg-Universität Mainz Duesbergweg 10–14 55128 Mainz Germany; ^2^ Department of Physics and Astronomy The University of Sheffield Western Bank Sheffield S10 2TN UK; ^3^ Department of Chemistry and Hylleraas Centre for Quantum Molecular Sciences University of Oslo PO-Box 1033 Blindern 0315 Oslo Norway; ^4^ Department of Organic Materials Science Yamagata University 4-3-16 Jonan Yonezawa Yamagata-ken 992-8510 Japan

**Keywords:** detergents, dimers, micellles, molecular modeling, self-assembly

## Abstract

The shape and size of self‐assembled structures upon local organization of their molecular building blocks are hard to predict in the presence of long‐range interactions. Combining small‐angle X‐ray/neutron scattering data, theoretical modelling, and computer simulations, sodium dodecyl sulfate (SDS), over a broad range of concentrations and ionic strengths, was investigated. Computer simulations indicate that micellar shape changes are associated with different binding of the counterions. By employing a toy model based on point charges on a surface, and comparing it to experiments and simulations, it is demonstrated that the observed morphological changes are caused by symmetry breaking of the irreducible building blocks, with the formation of transient surfactant dimers mediated by the counterions that promote the stabilization of cylindrical instead of spherical micelles. The present model is of general applicability and can be extended to all systems controlled by the presence of mobile charges.

## Graphical abstract

Micellar shape changes of sodium dodecyl sulfate are associated with different binding modes of the counterions. Morphological changes are caused by symmetry breaking of the irreducible building blocks, with the formation of transient surfactant dimers mediated by the counterions that promote the stabilization of cylindrical instead of spherical micelles.

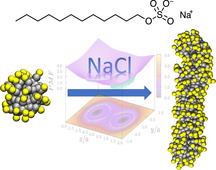

## Introduction

The optimal spatial organization of objects of a given shape, the so‐called *packing problem*, is an open question tantalizing the minds of scientists since several centuries. The most prominent example of hard spheres packing dates back to Kepler, and has been resolved only a couple of decades ago.^1^, ^2^, ^3^ In addition to the purely intellectual challenge, the solution to such a problem is a key to several phenomena in condensed matter physics, examples including the morphology of atomic and molecular crystals,^4^, ^5^ the folding of proteins,^6^ or the self‐assembly of rigid bodies^7^ or semi‐flexible polymers in a confined space.^8^

Amphiphiles self‐assemble into an array of nanostructured aggregates with various geometries.^9^, ^10^, ^11^, ^12^ Prototypically, surfactants form spherical micelles, but they are also commonly known to assemble into elongated ellipsoids and cylindrical micelles as well as vesicular structures.^13^ The shape and size of molecular aggregates is critical for their properties and function, both in chemistry and biology where they, for example, play a key role in molecular recognition, signalling processes, and transport in biological events. For example, the size and shape are critical for templating nanoparticle synthesis,^14^ determining the tissue permeability of drug carriers,^15^, ^16^ controlling cellular vesicular trafficking,^17^, ^18^ as well as for protein‐crowding properties.^19^, ^20^

**Figure 1:**
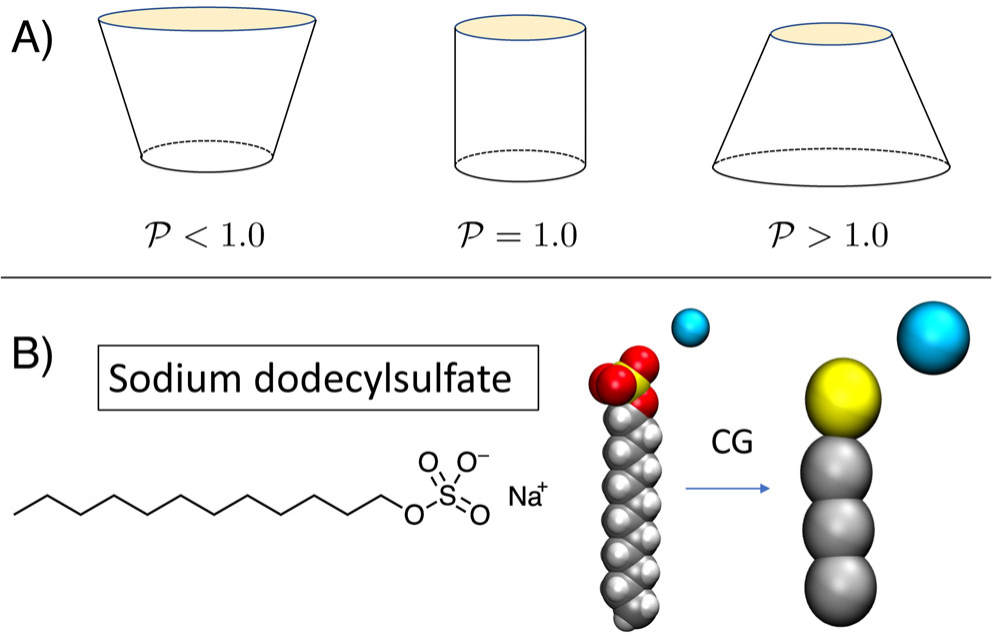
Panel A: Change of the packing parameter as a function of the ideal shape of self‐assembling objects. Panel B: Left: chemical formula of sodium dodecylsulfate. Right: coarse grained mapping of the surfactant used in this study.

The determination of the packing of surfactants contains additional challenging aspects compared to that of hard objects due to the soft nature of their boundaries. The geometrical shape of these structures is often rationalized in terms of a simple packing parameter 𝒫 defined by Israelachvili et al.^21^ as [Eq. (1)]:

(1) $$𝒫=\frac{v_{0}}{a_{0}\cdot \ell }$$

where *v*_0_ is the tail volume of the monomers, *a*_0_ the surface area of the micelle and ℓ is the length of the hydrophobic tail (Figure 1 A). It is commonly found that for low values of 𝒫 spherical structures are found, while values larger than about 1/2 favours cylinders, flat layers, or vesicles.

The concept of a molecular packing parameter has been widely used in chemistry, physics, and biology as it allows a simple and intuitive insight circumventing considerations about intermolecular forces.^11^, ^22^ However, the interactions driving the aggregation may not be always approximated by pure contact potentials. This is particularly true in the case of ionic amphiphiles, where the dominating intermolecular forces are constituted by long‐range Coulomb forces mediated by electrolytes. For example, very recently it has been shown that electrostatic screening can induce dramatic morphological changes in peptide/amphiphile aggregates on a micrometer scale.^23^ Similarly, electrostatic interactions have been indicated as the cause for the appearance of Franck‐Kasper quasi‐crystal phases in ionic surfactants.^24^ In such cases, a more reliable predictive understanding of the micellar structure requires a theoretical model taking into account the free energy contribution including all local and non‐local energy terms responsible for hydrophobic tail packing or hydrophilic head‐head repulsion, as well as the translational entropy of surfactant monomers, aggregates and importantly, the counterions.

Sodium dodecylsulfate (SDS, Figure 1 B) provides an archetypical example of the complexity of the packing problem when dealing with ionic surfactants. SDS is one of the most studied amphiphiles due to its wide array of applications in science and industry, finding use as a detergent in personal hygiene products such as tooth paste or shampoo^25^ or as an extraction tool for protein and DNA (SDS‐PAGE^26^). As suggested by its simple molecular structure, at low concentrations above the critical micellar concentration (cmc) ≈8 mm), SDS assembles into regular small spherical micelles of ≈2–3 nm in radius. In fact, for concentrations much larger than the cmc, the micelles undergo a morphological transition, transforming into cylindrical structures spanning lengths on the order of ≈100–1000 nm.^12^ Moreover, both experiments^27^, ^28^, ^29^, ^30^ and simulation studies^31^, ^32^, ^33^ show that such a transition can be drastically enhanced by the presence of a significant excess of salts in the solution. The physical origin of such a behavior may be attributed to non‐linear electrostatic effects involving both the surfactants, the free ions, and the solvent, as also suggested by all‐atom molecular dynamics simulation studies on pre‐micellar aggregates.^34^

In this work, we elaborate on the microscopic mechanism that drives the morphological transition in charged surfactant micelles by using a combination of molecular simulations, toy models and scattering techniques. We show that counterion binding can break the local symmetry of the assembly by pairing two SDS molecules. The change in shape into cylindrical micelles is thus promoted by a critical concentration of such lower‐symmetry moieties without any discontinuity in the overall packing parameter.

## Results and Discussion

### Morphological Transitions Can Be Tracked by SAXS/SANS

The structural transitions in SDS were mapped experimentally using small‐angle X‐ray/neutron scattering (SAXS/SANS) which provides the relevant structural information on length scales of about 1–100 nm. The data were analysed on an absolute scale using well established core–shell models for spherical/ ellipsoidal, cylindrical and worm‐like micelles^28^, ^35^ (See SI for more details).

**Figure 2:**
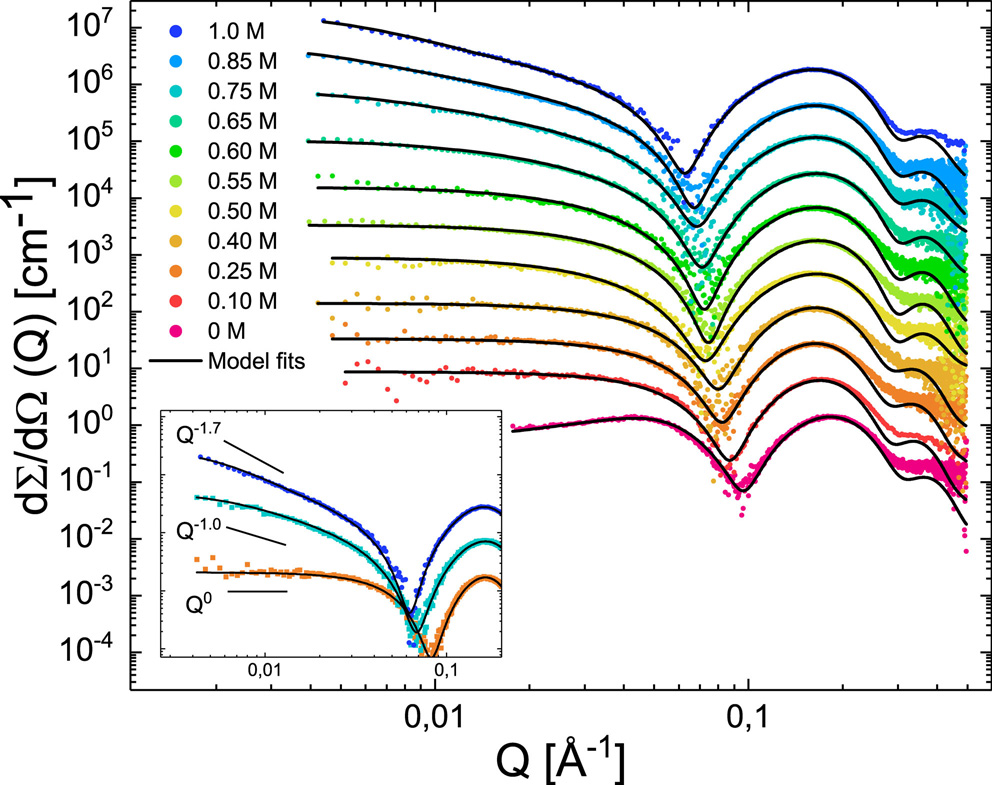
SAXS data for 43 mm solutions of SDS at various amounts of salt (0 m to 1.0 m). For clarity, the data has been shifted vertically with factors 4^*n*^ where *n* goes from 0 to 10 from bottom to top. The solid lines display fits of the quantitative scattering models (see text for detail). The inset shows selected scattering curves from the main figure (0.25 m, 0.75 m and 1.0 m) together with lines corresponding to the theoretical scattering behaviour of small ellipsoidal or cylindrical micelles (*Q* ^0^), long cylindrical micelles (*Q* ^−1^) and worm‐like micelles (*Q* ^−1.7^). All data is at 37 °C.

In a first set of experiments, we explored the existence of a transition in low concentrations of SDS and increasing concentrations of NaCl. Figure 2 shows the SAXS scattering intensity as a function of the modulus of the scattering vector $Q=\frac{4\pi \sin(\theta /2)}{\lambda }$ , where *λ* is the X‐ray wavelength and *θ* is the scattering angle, for a 43 mm concentration of SDS. At zero or low concentrations of salt, the SAXS scattering pattern on a double logarithmic scale show a peak or almost flat intensity at low *Q* which indicates predominantly spherical or spheroidal micelles with repulsive interactions. However, at higher salt concentrations, above 0.4–0.5 m NaCl, the intensity at low *Q* increases drastically and follows a steeper *Q* ^−1^ decay and, upon even more salt added, we find a ≈*Q* ^−1.7^ dependence (Figure 2, inset). This demonstrate a transformation into cylindrical‐like or worm‐like micelles, respectively, in agreement with previous studies.^27^, ^28^, ^30^, ^35^ The latter suggests the formation of flexible worm‐like micelles that, below the Kuhn length are cylindrical, but at a more global scale (lower *Q*) resemble chain‐like structures. These micelles increase their contour length with increasing concentration of salt, reaching up to fractions of a μm for salt concentrations around 1 m.^36^ The same features are found for all concentrations in the dilute range investigated by SAXS (See fit results in the Supplementary Information (SI), Figure S1 and Tables S3–S5).

The spherical to cylindrical morphological transition was already suggested several decades ago by Reiss and Luzzatti^37^ to occur in salt‐free solutions but at very high concentrations (≈1 m). This transformation was qualitatively reproduced by multiple computational studies using different modelling approaches.^31^, ^32^, ^33^ However the location of the transition was found at lower concentrations (≈0.3–0.4 m). Here, we performed SANS experiments which are well suited to study highly viscous samples, to detect the transition in a very broad concentration range of SDS (0.26 m to 1.87 m). At low concentrations, our data clearly detect regular spherical micelles, as expected. Strikingly, at a concentration of 1.04 m, the shape of the SANS intensity shows a progressive broadening that cannot readily be described by a single morphological population (see Figure S3). This indicates the co‐existence of SDS micelles of different morphologies. However upon increasing the SDS concentration further to 1.87 m and above, we find a crystalline hexagonal phase formed by well‐packed cylindrical micelles giving rise to Bragg‐like reflections as seen in Figure S3. This is clearly visible from the three reflections positioned at *Q*_m_, the value of *Q* at the first peak maximum intensity, $\sqrt{3}Q_{m}$ and 2*Q*_m_, respectively.

### Computer Simulations Reproduce the Micellar Shape Transitions

The relationship between the change in the morphology of SDS micelles and the total concentration of electrolytes was investigated by a set of hybrid particle‐field/molecular dynamics (hPF‐MD)^38^, ^39^, ^40^ computer simulations at different SDS and salt concentrations, using the same protocol as in ref. 33, which reproduced the qualitative trends observed in the experiment.

**Figure 3:**
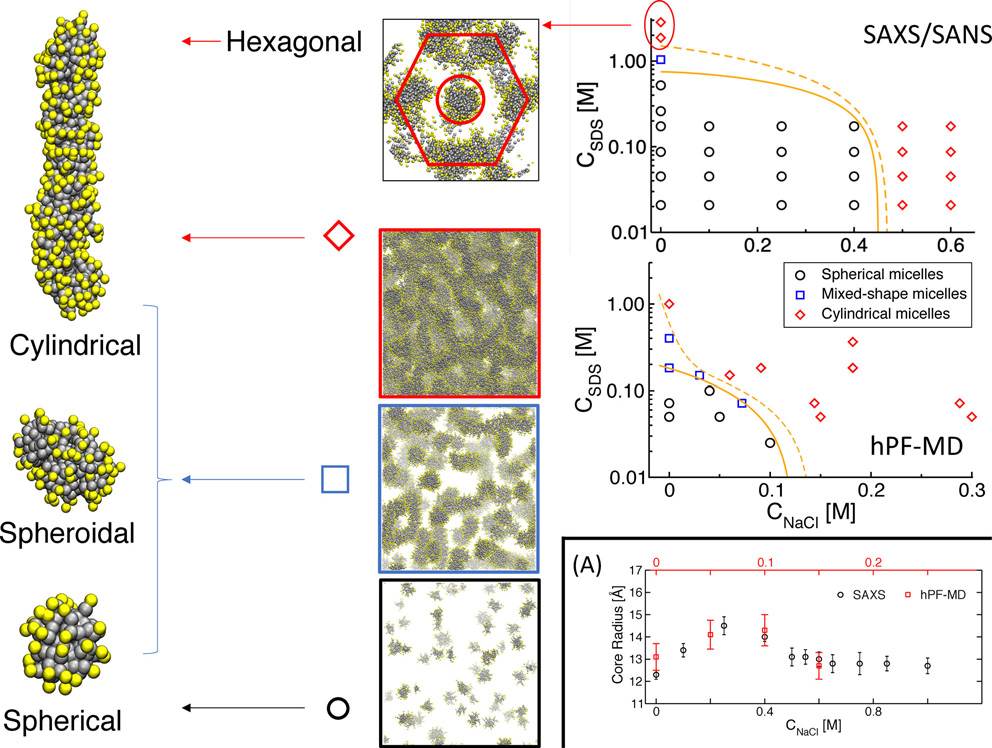
Structures of SDS micelles as a function of the concentration of SDS and salt from SAXS/SANS data, and hPF‐MD simulations. The squares reproduce representative snapshots from simulations. The area between the orange lines in the right plot represents the transition region where both spheroidal and cylindrical micelles are observed. **Panel A**: core radius of the SDS micelles in a 0.045 m solution as a function of the salt concentration. Data from experiment (black) and hPF‐MD simulations (red).

At low SDS concentrations, the hPF‐MD data reproduce the presence of well‐defined small spherical micelles that transform into cylindrical aggregates by addition of an excess of salt into the system. Although the critical salt concentration for the conformational transition is quantitatively underestimated by hPF‐MD, the observed trend for the core radius of the micelle is in very good agreement with the experiment (Figure 3 (A)). In all cases, the values for the principal core radius of the ellipsoidal micelles are around 13–14 Å which is very close to the molecular contour length (14.1 Å), indicating that the SDS molecules always remain in a straight conformation. Above the transition, the core radius of the worm‐like micelles relaxes to 12–13 Å. In accord with experiment, the contour length of the cylindrical micelles near the transition region is ≈80±13 Å, and rapidly extends to lengths exceeding the size of the simulation box.

Increasing the surfactant concentration in pure SDS solutions, the hPF‐MD simulations predict the formation of cylindrical objects above a salt concentration of 0.2 m, in agreement with previous computational models.^31^, ^32^, ^33^ Nonetheless, in this case the transition is not sharp, and even at double concentration the solution contains heterogeneous micellar aggregates with spherical, spheroidal, and cylindrical shapes (Figure 3). At 1 m concentration the SDS micelles are almost only cylindrical with very long contour lengths organised in a disordered network. Overall, as seen in (Figure 3), the simulated phase diagram follows the qualitative trends found in the experiments. In particular, both experiment and simulations report a diffuse region of coexistence between spherical and cylindrical shapes in the absence of excess salt, while the transition becomes relatively sharp in saline solution. The quantitative discrepancy between simulations and measurements should be attributed to the approximations in the model, most importantly the evaluation of intermolecular interactions in the mean‐field limit, and the use of a constant dielectric value, as previously reported.^33^ The hexagonal arrangement observed at >2 m concentrations (Figures 3, S2) is also in agreement with the regular hexagonal crystalline phase recently observed in coarse‐grained simulations.^41^

### Salt Localization on the Micelle Signals the Morphological Transition

Repeating hPF‐MD simulations over a broad range of SDS and salt concentrations, we observed that the critical SDS concentration at which cylindrical aggregates are detected decreases linearly with addition of salt (Figure 3). Interestingly, regardless of the concentration of SDS the transition from small spheroidal micelles to elongated structures occurs at a small value for the packing parameter (𝒫≈0.2 ) and does not show an discontinuity at the morphological transition. The threshold value is much smaller than what was proposed by Israelachvili (𝒫≥0.5 ). This implies that such a transition in ionic surfactants cannot be related to the pure molecular dimensions as found for non‐ionic surfactants.^42^

For a better understanding of the role of counterions in the morphological transition, we introduce the order parameter *ξ*_mic_ defined as [Eq. (2)]:

(2) $$\xi_{mic}=\frac{1}{2\langle {\mathrm{Na}}^{+}\rangle }\int_{A}\left|\sigma_{{\mathrm{Na}}^{+}}\left(r\right)-\frac{\left\langle {\mathrm{Na}}^{+}\right\rangle }{A}\right|dA$$

where $\left\langle {\mathrm{Na}}^{+}\right\rangle$ is the average number of ions bound on the surface of a micelle, *A* is the surface area of the micelle, and $\sigma_{{\mathrm{Na}}^{+}}\left(r\right)$ is the local surface density of ions. *ξ*_mic_ compares the local density of Na^+^ on the surface of the micelle to the average surface density value. High values of *ξ*_mic_ correspond to counter‐ions bound in localized areas of the surface of the micelle (i.e., in the proximity of individual SDS heads). Low *ξ*_mic_ values indicate that the ions are homogeneously dispersed onto the whole surface of the micelle.

**Figure 4:**
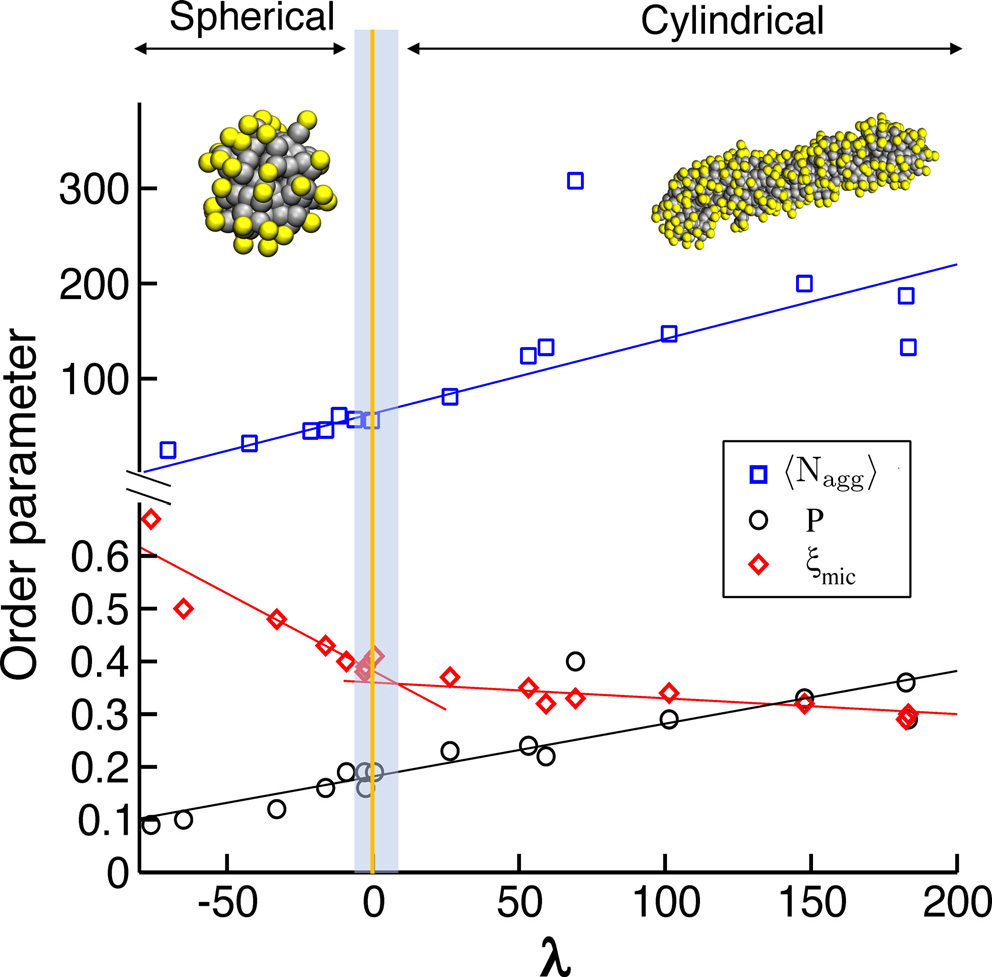
Order parameters for the sphere‐to‐cylinder transition. The *x* axis reports the reaction coordinate *λ*, defined as the distance of a system at specific SDS/salt concentration conditions from the orange transition full‐line in Figure 3. Negative values of *λ* indicate the region of stability for small spherical micelles, positive values indicate concentrations of SDS and salt at which cylindrical micelles are detected. The plot reports the average SDS aggregation number $\left\langle N_{\mathrm{agg}}\right\rangle$ (blue squares), the average packing parameter 𝒫 (black circles), and the average salt delocalization *ξ*_mic_ (red diamonds).

Computational data report a clear change in *ξ*_mic_ corresponding to the morphological transformation of the aggregates (Figure 4). In particular, the spherical micelles are characterized by more localized counterions with higher values of *ξ*_mic_. The closer the systems are to the critical transition area (Figure 3), the more ions bind in a delocalized manner. The transition to cylindrical aggregates is signalled by a discontinuity in the derivative of *ξ*_mic_. The value of *ξ*_mic_ remains rather constant in all cylindrical aggregates regardless of the distance from the transition region (Figure 4).

### A Simple Electrostatic Model Explains the Change in Molecular Packing

**Figure 5:**
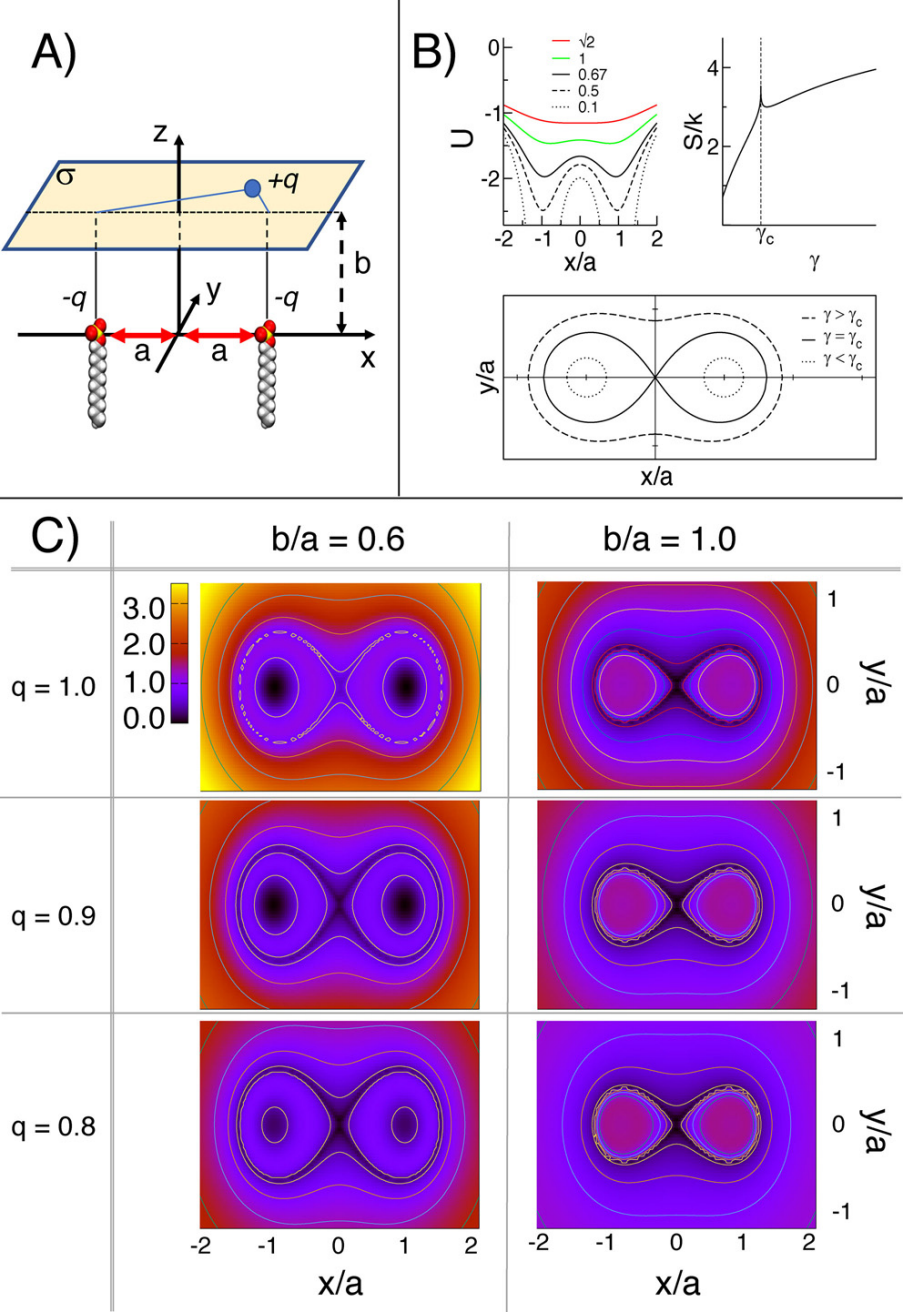
**Panel A**: Toy model composed by a charge *q* moving on a surface *σ* located at a height *b* from two charges with same, opposite sign, distant 2*a* from each other. **Panel B**: Top‐left plot: Binding energy of the mobile charge along the *y=*0 axis of the *σ* plane (in generalised units $U=-{\left(2\gamma \right)}^{-1}$ ). Top‐right plot: Entropy of the mobile charge as a function of *γ* at a fixed *b*/*a* ratio. *Bottom panel*: Isoenergetic contour lines on *σ* for *γ*<*γ*_c_ (dotted line), *γ*=*γ*_c_ (continuous line), and *γ*>*γ*_c_ (dashed line). **Panel C**: Map of the potential of mean force for the mobile charge on *σ* for different values of *b/a* and *q*. Values are given as multiples of *k*_B_ *T*/*a*.

To understand the behaviour of the Na^+^ ions bound to the surface of the SDS micelles, we introduce a toy‐model formed by one ideal point charge +*q* moving on the plane *σ* parallel to the *xy* plane at a distance *b*, in the presence of two negative charges −*q* fixed at a relative distance 2*a* along the *x* axis (Figure 5 A). In this simple system, the negative charges represent two SDS heads aggregated with an equilibrium head‐to‐head distance of 2*a*, while *σ* is a simplified flat representation of the solvent‐accessible surface over which a counterion is bound.

The binding energy *U* of the positive charge, expressed as a function of the distances (*R*_1_,*R*_2_) from the two negative charges, is [Eq. (3)]:

(3) $$U\left(R_{1},R_{2}\right)=-\frac{q^{2}}{4\pi \epsilon }\left(\frac{1}{R_{1}}+\frac{1}{R_{2}}\right),$$

which can be rewritten as [Eq. (4)]:

(4) $$a\left(\frac{1}{R_{1}}+\frac{1}{R_{2}}\right)=\frac{1}{\gamma }$$

where we have introduced $\gamma =-\frac{q^{2}}{4\pi \epsilon }\frac{1}{aU}$ , a reduced length analogous to the Bjerrum length. For *b*/*a*<√2, the binding energy *U* exhibits two minima in correspondence of the SDS heads (Figure 5 B). The binding mode is determined by the critical value $\gamma_{c}=\frac{1}{2}\sqrt{1+{\left(\frac{b}{a}\right)}^{2}}$ . In fact, for *γ*<*γ*_c_, corresponding to stronger binding energies, isoenergetic lines on *σ* encircle the individual poles; thus, the bound charge localizes around one of the two SDS heads (Figure 5 B). On the contrary, for *γ*≥*γ*_c_ isoenergetic lines surround both poles, implying delocalized binding. At *b*/*a*=√2 the two minima of *U* coalesce at (0,0); as a consequence, for *b*/*a*≥√2 localization of the mobile ion above either one of the two negative charges cannot occur.

The entropy *S*(*γ*) of the bound charge at a fixed energy is expressed as [Eq. (5)]:

(5) $$S\left(\gamma \right)=k_{B}lnℒ\left(\gamma \right)+C\left(a,b\right)$$

where $ℒ\left(\gamma \right)$ is the length of the isoenergetic contour line on the *σ* plane that solves Equation (4) at a given *γ*, and *C* is a constant that depends on the *a*, *b* parameters only. At constant temperature, *C* can be disregarded as it only produces a constant shift in the free energy scale and thus does not affect the minimization of the function. Interestingly, *γ*_c_ corresponds to a local maximum cusp in *S*(*γ*) (Figure 5 B).

From the definitions of *U*(*γ*) and *S*(*γ*) we can express the mean‐field free energy, or potential of mean force, $ℱ\left(\gamma \right)$ on *σ* [Eq. (6)]:

(6) $$ℱ\left(\gamma \right)=-\frac{q^{2}}{4\pi \epsilon a}\frac{1}{\gamma }-k_{B}Tlnℒ\left(\gamma \right)$$

Figure 5 C reports $ℱ\left(x,y\right)$ of the free charge on *σ* as a function of the *b*/*a* ratio, and the charge *q*. For *b*/*a*<√2, the free energy map shows qualitatively the same topological features, regardless of the values for *b*/*a* and *q*. Specifically, ℱ is characterized by two degenerate minima localized on the *x* axis, corresponding to a localized binding of the mobile charge over one of the two negative poles (*M*_l_). These sites are associated to the contribution of *U* to ℱ , as they correspond to the global minimum of the electrostatic energy. One additional minimum for ℱ (*M*_d_) resides in a delocalized region comprising the midpoint (0,0) and a broader area around the two *M*_l's_. The origin of *M*_d_ is entropic, as it corresponds to a spatial region characterized by a maximum in the density of states.

As *U* is directly dependent on *q*, any change in *q* affects the free energy difference Δℱ between *M*_l_ and *M*_d_. In particular, as shown in Figure 5, for relatively small values of *b*/*a* and high values of *q*, the mobile charge binds preferentially to *M*_l_. Reducing the absolute value of *q*, the free energy difference between *M*_l_ and *M*_d_ decreases until *M*_d_ becomes the preferential binding mode. The critical value at which the change in binding behaviour occurs is related the binding energy difference Δ*U* between the two modes, which depends on *b*/*a*. In fact, while small values of *b*/*a* favour localized binding, larger values of such ratio promote binding to *M*_d_. At the critical value of *b*/*a*=√2 the minimum of *U* corresponds geometrically to *M*_d_, making it the only possible binding mode.

Albeit very simple, this model is able to provide a clear qualitative explanation of the change in *ξ*_mic_ along the morphological transition. At low SDS/salt concentrations, thus in the absence of a strong electrolyte screening, and at low concentration of SDS (low *b*/*a* ratio) Na^+^ ions preferentially bind to individual SDS heads (*M*_l_), evidenced by high *ξ*_mic_ values. The addition of free electrolytes in the solution screens the charge‐charge interactions, effectively flattening the depth of (*M*_l_), and producing a decrease in *ξ*_mic_. An equivalent effect is obtained by increasing the concentration of SDS, as the crowding of the molecules reduces the average head‐to‐head distance, increasing the *b*/*a* ratio. Past the critical line, ions preferentially bind in a delocalized manner (*M*_d_). As this type of binding is dominated by the entropic contribution, the *ξ*_mic_ remain practically unaffected by ulterior addition of free electrolytes or SDS.

Although the basin of *M*_d_ corresponds to a large region distributed around the two SDS heads, most of the binding region is localized around the midpoint between the negative charges. The anisotropic distribution of the counterions suggests that the head‐to‐head distance between paired SDS heads is shorter than that with other SDS neighbours. A shorter average head‐to‐head distance along the longitudinal axis of the cylindrical micelles (6.2 Å against 8.6 Å, Figure S4) suggests that supramolecular dimers would align in that direction.

### Mechanism of the Transition from Spherical to Cylindrical Micelles

The spherical organisation of single‐chain surfactant micelles is commonly interpreted as the packing of molecules of effective conical shape, where the hydrophobic carbon chain correspond to the axis of the cone, and the hydrophilic head resides at the center of the base of the cone. The relatively larger radius of the conical basis is a consequence of the volume occupied by the water molecules hydrating neighboring heads. In the case of electrolytic compounds like SDS, any understanding based on packing consideration requires proper inclusion of the mobile counter‐ions. In particular, both the experiment and previous simulations show that addition of salt into a SDS solution facilitates a drastic morphological change in the shape of the SDS micelles, implying that the presence of additional mobile charges directly affects the shape of the packing objects.^27^, ^30^, ^31^, ^32^, ^33^

**Figure 6:**
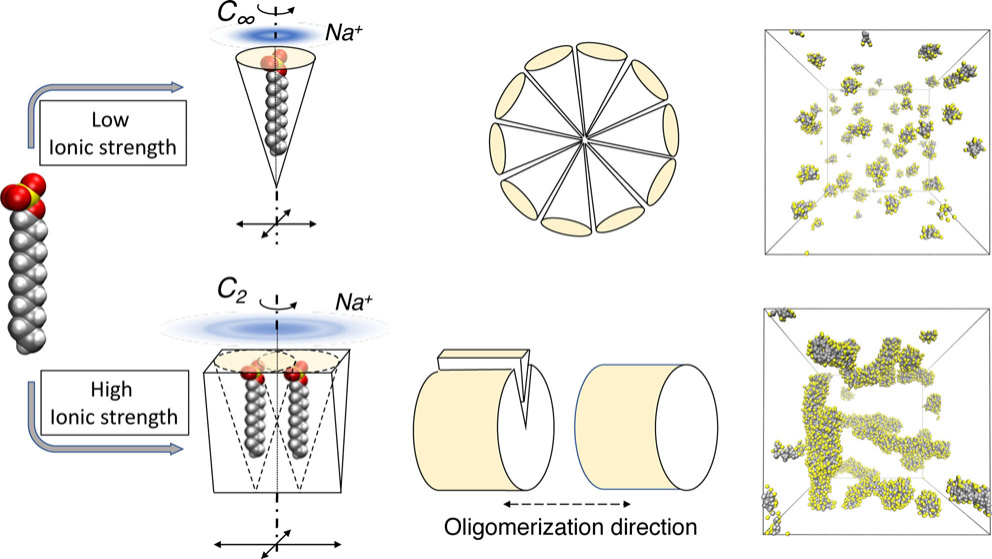
Mechanism of SDS self‐assembly. Salt‐induced changes in the symmetry of SDS unit elements leads to different assembly routes, yielding aggregates of different shapes.

The constitution of supramolecular irreducible units formed by at least two SDS molecules gives rise to the change in the structure of the SDS micelles. In the case of a low sodium concentration each individual SDS molecule behaves like independent objects each of roughly *C*_∞*v*_ symmetry. Thus, in a self‐assembled structure, SDS would occupy an effective volume of a (truncated) cone, with the larger base at the height of the repulsive, hydrophilic, negatively‐charged sulfate head, and a lateral hydrophobic surface. Conical objects that pack along the sides assemble into spherical aggregates, which is in fact the shape of SDS micelles at low ionic strength (Figure 6).

Concomitant binding of Na^+^ over multiple SDS molecules determines the existence of lower‐symmetry (*C*_2*v*_) supramolecular SDS units characterized by an effective shape similar to that of a wedge. Remarkably, the estimate value for the packing parameter for such object is 𝒫=0.493 , practically at the threshold value of 1/2. Intuitively, such shapes can build up in disc‐like structures, which can grow longitudinally to form long cylindrical self‐assembled objects. This implies an anisotropic distribution of SDS heads, with more packed heads along the longitudinal axis of a tubular micelle than along the transversal one, consistent with what was found in our simulations (Figure 6). Notably, as SDS is a strong electrolyte, a large part of the counter‐ions disperse in the solvent, leaving the SDS micelle negatively charged. In fact, both simulation and experiment indicate that 60–75 % of SDS molecules are screened by counterions. Moreover, by a weighted average of 𝒫 for monomeric and dimeric SDS, we estimate that ≈10 % of the micellar charge is balanced by ions bound to SDS dimers at the sphere/cylinder transition. These data indicate that only ≈20 % of the SDS are required to pair in the more compact dimeric form to induce the structural change of the micelle. Thus, the change in morphology is promoted by the statistical presence of a critical number of more packed, lower‐symmetry units that dynamically form and break along the micelle, according to the binding dynamics of the counterions.

## Conclusion

The work presented here contributes to the understanding of the self‐assembly of soft matter in the presence of dominant long‐range interactions. In particular, it evidences how, in the right conditions, such forces can stabilize supramolecular entities. The symmetry breaking produced by such moieties induces a different local organization of the assembling molecules, and consequently a different global shape of the macroscopic structure. In these cases, reasoning in term of close molecular packing still holds if applied at the supramolecular scale, taking into consideration not simply the shape of the individual molecule, but the statistical presence more complex organization.

Understanding the effect of long‐range interactions on the morphology of charged surfactants is of general relevance for biological and chemical systems. A prominent example is provided by the outer cell wall of Gram‐ bacteria, which is composed by strongly negatively‐charged lipopolysaccharides. The stability of the lamellar phase of such lipids is critically determined by the interaction with specific counterions.^43^, ^44^, ^45^, ^46^ Being able to predict how lipid‐ion interactions determine membrane properties can be crucial for the development of new effective antibiotics, as different bacterial lipid chemotypes are able to modulate the response of the membrane to the binding of positively‐charged drugs.^47^ In general, transport processes, trafficking and cellular functions and interactions are often critically dependent on the curvature and effective charges, as observed in membranes consisting of complex mixtures of various charged, zwitterionic and neutral lipids and proteins.^48^, ^49^ The stability and dynamic processes of lipid vesicles and lipoproteins are also controlled by electrostatic interactions,^50^, ^51^ which can play a key role in the use of amphiphiles as nano‐reactors, where they not only control the morphology but also influence the kinetic pathways of chemical reactions.^52^

So far, the packing problem of surfactants has typically been addressed in terms of the shape of the aggregating molecules only, although attempts have been made to take into accounts more detailed interactions^22^ and also the contribution from counterions.^53^ Sangwai et al.^53^ report that ions are weakly bond to the charged head groups and form an electrical bilayer around the micelle‐water interface. This screens the intra‐micellar electrostatic repulsion which leads to a smaller surface area per surfactant and consequently to the sphere to cylinder transition. Here, we identified the critical importance of the counterion, not only for the quantitative modulation of the forces driving the micellar formation, but also in determining the shape of the aggregating units. It is worth mentioning that accurate models for aggregation of charged surfactants need to also take into account ion specificity, which, by influencing the binding affinity of ions characterised by the same charge, can quantitatively produce differences in the aggregation state at the same thermodynamic conditions.^46^, ^54^ The role of free ions in the organization of the surfactants has been only poorly understood so far, and the general model proposed in this work provides a route toward accurate investigations of such effects. Experimental detection of the found pairing mechanism would be challenging using static scattering experiments given the small fraction of bound amphiphiles (≈20 %) and the expected low signal/noise at high *Q*. However, we speculate that dynamics methods, for example, inelastic scattering techniques or NMR techniques could provide experimental insight into this mechanism.

### Supporting material

Computational and experimental section, additional SAXS spectra at different concentrations of SDS and NaCl, SANS spectra at different concentrations of SDS, head‐to‐head distributions in spherical and cylindrical micelles.

### Acknowledgement

This work was supported by the Research Council of Norway (RCN) through the CoE Hylleraas Center for Quantum Molecular Sciences (Grant n. 262695) and by the Norwegian Supercomputing Program (NOTUR) (Grant n. NN4654K). KS, GD, JG, and MC acknowledge funding by the Deutsche Forschungsgemeinschaft (DFG) within the projects B3 and B5 of the TRR 146 (project number 233630050). HBK received funding from the European Union Horizon 2020 research and innovation program under the Marie Skłodowska‐Curie Grant Agreement HYPERBIO—No 704491.

## Conflict of interest

The authors declare no conflict of interest.

## Supporting information

As a service to our authors and readers, this journal provides supporting information supplied by the authors. Such materials are peer reviewed and may be re‐organized for online delivery, but are not copy‐edited or typeset. Technical support issues arising from supporting information (other than missing files) should be addressed to the authors.

Supplementary
