# Supplementary material for: Supramolecular Packing Drives Morphological Transitions of Charged Surfactant Micelles
Source: Angew Chem Int Ed Engl. 2020 Aug 17;59(42):18591–8. doi: 10.1002/anie.202004522 (PMC7589243; doi:10.1002/anie.202004522)
Supplement: Supplementary file 1 — Supplementary [file ANIE-59-18591-s001.pdf]

Angewandte Chemie
Eine Zeitschrift der Gesellschaft Deutscher Chemiker

Supporting Information

Supramolecular Packing Drives Morphological Transitions of Charged Surfactant Micelles

Ken Schäfer, Hima Bindu Kolli, Mikkel Killingmoe Christensen, Sigbjørn Løland Bore, Gregor Diezemann, Jürgen Gauss, Giuseppe Milano, Reidar Lund, and Michele Cascella**

anie_202004522_sm_miscellaneous_information.pdf

Computational Methods

Hybrid particle-field MD

The SDS/salt/water molecular system was mapped into a coarse grained representation as in Figure S1. Multiple systems with different compositions (Table S1) were prepared by randomly placing the molecular components in a periodic cubic box of edge 33.2 nm. The total densities of the various systems correspond to an external pressure of $\sim$1 bar. For each system, we ran 5 μs of hPF-MD simulations in the *NVT* ensemble to obtain well equilibrated aggregate structures. We employed a time-step of 0.03 ps and a temperature of 298 K employing the Andersen thermostat [55] with a collision frequency of 7 ps^{-1}. Particle densities and charges were computed on a 50x50x50 mesh grid. The hPF parameters for the potential energy were taken from ref. [Main-text: 33]. hPF-MD simulations were performed using the OCCAM code (ref. [Main-text: 39]). Ref. [Main-text: 33] also contains a detailed explanation of the hPF-MD method with electrostatics, and its implementation in OCCAM.

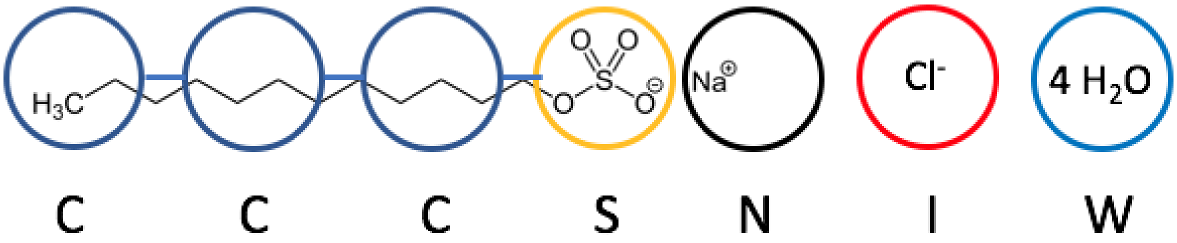

Figure S1: Schematic sketch of the coarse-grained model for the SDS molecule, water and sodium chloride. The sulfate head group is mapped into a single bead (S) and the hydrophobic tail into 3 beads (C). 1 water bead (W) represents 4 water molecules. The sodium (W) and chloride (I) ions also have their own bead types.

Table S1: Number of particles in the different simulation setups.

SDS + Nacl / mM	N_{total}	N_{SDS}	N_{Na^+}	$N_{\text{H}_2\text{O}}$	N_{Cl^-}
25 + 100	310028	503	2514	305000	2011
50 + 50	310840	1120	2240	303000	1120
50 + 150	313320	1120	4480	301000	3360
50 + 300	317040	1120	7840	298000	6720
72 + 72	311200	1600	3200	300000	1600
72 + 144	313400	1600	4800	299000	3200
72 + 288	317040	1600	8000	296000	6400
100 + 40	311537	2011	2816	300000	805
150 + 30	312288	3016	3620	296000	604
150 + 60	312494	3016	4223	295000	1207
182 + 91	317000	4000	6000	293000	2000
182 + 144	318000	4000	8000	290000	4000
182 + 288	323000	4000	12000	287000	8000

Experimental Section

Small-angle X-ray/neutron scattering

SAXS experiments were performed at the bio-SAXS BM29 beamline at the European Synchrotron Radiation Facility (ESRF) in Grenoble, France, with a detector distance of 2.87 meter and energy of 12.5 keV, covering a Q range of 0.0047 $Å^{-1}$ to 0.5 $Å^{-1}$ at 37 °C. SANS data were collected at the time-of-flight small-angle neutron scattering instrument Sans2d at the ISIS Neutron and Muon source, UK, using a single detector distance of 4m covering Q = 0.005 - 0.7 $Å^{-1}$.

Materials and sample preparation

Sodium Dodecyl Sulphate (> 99.0 %) and NaCl (> 99.9 %) were purchased from Sigma-Aldrich and VWR, respectively, and used as received. The solutions were freshly prepared prior to the measurements using Milli-Q water or deuterium oxide (99.9 atom %) from Sigma-Aldrich.

Scattering models

The model fitting was done on an absolute scale with molecular constraints and using known concentrations of the system using well-established core-shell models [Main-text: Refs. 28, 30]. The contrasts for the surfactant molecule were obtained from the scattering lengths densities ρ_i of the tail group, head group and solvent. For the head and tail, the scattering length density is calculated as $\rho_i = \sum_{i=1}^{N} b_i/V_i$ where b_i is the scattering length and V_i is the volume of the species. Using X-rays, the scattering lengths are given as $b_i = Z_i r_e$ where Z_i is the number of electrons and r_e is the scattering length of an electron (Thomson radius). The core and head group contrasts were calculated using literature values for the specific volumes of a C_{12}-chain [56] and the SDS molecule [57]. Moreover, an ionic dissociation degree of 27 % was assumed [57]. The head group volume V_{head} was optimized for each salt concentration to account for changes in the head group density and/or solvation layer affected by salt. The total intensity in the absence of interactions is given by:

$$I(Q) = n_z P(Q) \tag{S1}$$

Ellipsoid fit model

An ellipsoid of revolution with a core-shell structure has two minor core radii R_{core} and a major axis ϵR_{core}, in addition to a shell thickness t_{shell}. The radii for the outer shell ellipsoid are thus given as $R_{tot} = R_{core} + t_{shell}$ and $R_{tot} = \epsilon R_{core} + t_{shell}$, respectively, with an aspect ratio $\epsilon_{out} = (\epsilon R_{core} + t_{shell})/(R_{core} + t_{shell})$. The core volume is then $V_{core} = (4/3)\pi\epsilon R_{core}^3$, and

the total volume is $V_{tot} = (4/3)\pi\epsilon R_{tot}^3$. The analytical form factor is finally given as [58]

$$P_{cs,ell}(Q) = \int_0^{\pi/2} [\Delta\rho_{shell} V_{tot} A_{sph}(Qr_{tot}) + (\Delta\rho_{core} - \Delta\rho_{shell}) V_{core} A_{sph}(Qr_{core})]^2 \sin\,\alpha\, d\,\alpha \tag{S2}$$

with $r_i(\sin^2\alpha + \epsilon_i^2 \cos^2\alpha)^{1/2}$ and $A_{sph}(x) = 3[\sin\,x - x\cos\,x]/x^3$ [59].

To account for inter-particle interactions at high surfactant concentrations, the Hayter-Penfold structure factor [60] was applied. The structure factor is controlled by the hard-sphere radius R_{HS}, the number of charges per particle Z and the hard-sphere volume fraction η. If the effective charge is set to zero, the potential reduces to the well-known Percus-Yevick hard core potential.

Cylinder fit models

Cylindrical core-shell micelles have a core radius R_{core}, a total radius $R_{tot} = R_{core} + t_{shell}$ where t_{shell} is the shell thickness, and a contour length of the core L_c. The volume of the core is given as $V_{core} = \pi R_{core}^2 L_c$, and the total volume is given as $V_{tot} = \pi R_{tot}^2 (L_c + 2t_{shell})$. The form factor is then [58]

$$\begin{aligned} P_{cs,cyl}(Q) = \int_0^{\pi/2} \Bigg[& \Delta\rho_{shell} V_{tot} \frac{2J_1(QR_{tot}\sin\,\alpha)}{QR_{tot}\sin\,\alpha} \frac{\sin(Q(\frac{1}{2}L_c + t_{shell})\cos\,\alpha)}{Q(\frac{1}{2}L_c + t_{shell})\cos\,\alpha} \\ & + (\Delta\rho_{core} - \Delta\rho_{shell}) V_{core} \frac{2J_1(QR_{core}\sin\,\alpha)}{QR_{core}\sin\,\alpha} \frac{\sin(Q(\frac{1}{2}t_{shell})\cos\,\alpha)}{Q(\frac{1}{2}t_{shell})\cos\,\alpha} \Bigg] \end{aligned} \tag{S3}$$

with $J_1(x)$ is the first order Bessel function of first kind.

To account for the flexibility of very long cylindrical micelles, the Kuhn length b can be introduced. It is only for $L_c > b$ that the micelles are flexible and so-called worm-like. $L_c < b$ corresponds to rod-like micelles. The form factor for such a core-shell structure is given by

$$P_{cs,worm}(Q) = [\Delta\rho_{shell} V_{tot} A_{cr}(QR_{tot}) + (\Delta\rho_{core} - \Delta\rho_{shell}) V_{core} A_{cr}(QR_{core})]^2 \, P_{chain}(Q, L_c, b) \tag{S4}$$

where A_{cr} is the scattering amplitude for a circular cross-section given by $A_{cs}(x) = 2J_1(x)/x$. $P_{chain}(Q, L_c, b)$ gives the scattering from the worm-like features in the longitudinal direction. It has been shown by analyzing scattering patterns of worm-like chains [61–63] that the form factor expression can be written as a combination of the scattering from a rod of length L_c [64], and from a random-walk self-avoiding chain with length L_c and Kuhn length b [62, 65]. To account for polydispersity in the

contour length L_c, the following average form factor was instead used

$$\langle P_{cs,worm}(Q)\rangle = \frac{\int_{2R_{core}}^{1000L_c} L_c^2 f(L_c) P_{cs,worm}(Q, L_c, b) dL_c}{\int_{2R_{core}}^{1000L_c} L_c^2 f(L_c) dL_c} \tag{S5}$$

An exponential distribution in size was used and given by $f(L_c) = \exp(-L_c/L_{c,mean})$, which is the expected equilibrium size distribution for worm-like micelles with an average length $L_{c,mean}$ [66]. The lower bounds of the integrals account for the minimum size of the micelles, while the upper bound is a value where the contributions becomes negligible.

For cylindrical and worm-like micelles, the structure factor is more complex as it involves both inter- and intra-chain interactions, and there are no analytical expressions available. There are still approximations available, one of them being an empirical structure factor from the PRISM theory which can be written as [67]

$$S(Q) = \frac{1}{1 + \beta c(Q, R') P_{rod}(Q, L_c - 2R)} \tag{S6}$$

where β is a fit parameter proportional to the excluded volume and

$$P_{rod}(Q, \ell) = \frac{2Si(Q\ell)}{Q\ell} - \frac{4\sin^2(Q\ell/2)}{Q^2\ell^2}. \tag{S7}$$

Where $Si(x) = \int_0^x \frac{\sin t}{t} dt$, and $c(Q)$ is the direct correlation function between sites on the rod where the size of the correlations holes is $2R'$, and can be approximated as

$$c(Q, R') = \frac{3(\sin(Q2R') - Q2R' \cos(Q2R'))}{(Q2R')^3}. \tag{S8}$$

Estimation of core radius The estimation of the core radius presented in Figure 3 (main text) was obtained by analysing the scattering patterns in the whole Q-range, from which both the local core-shell cross-section and the average length can be determined.

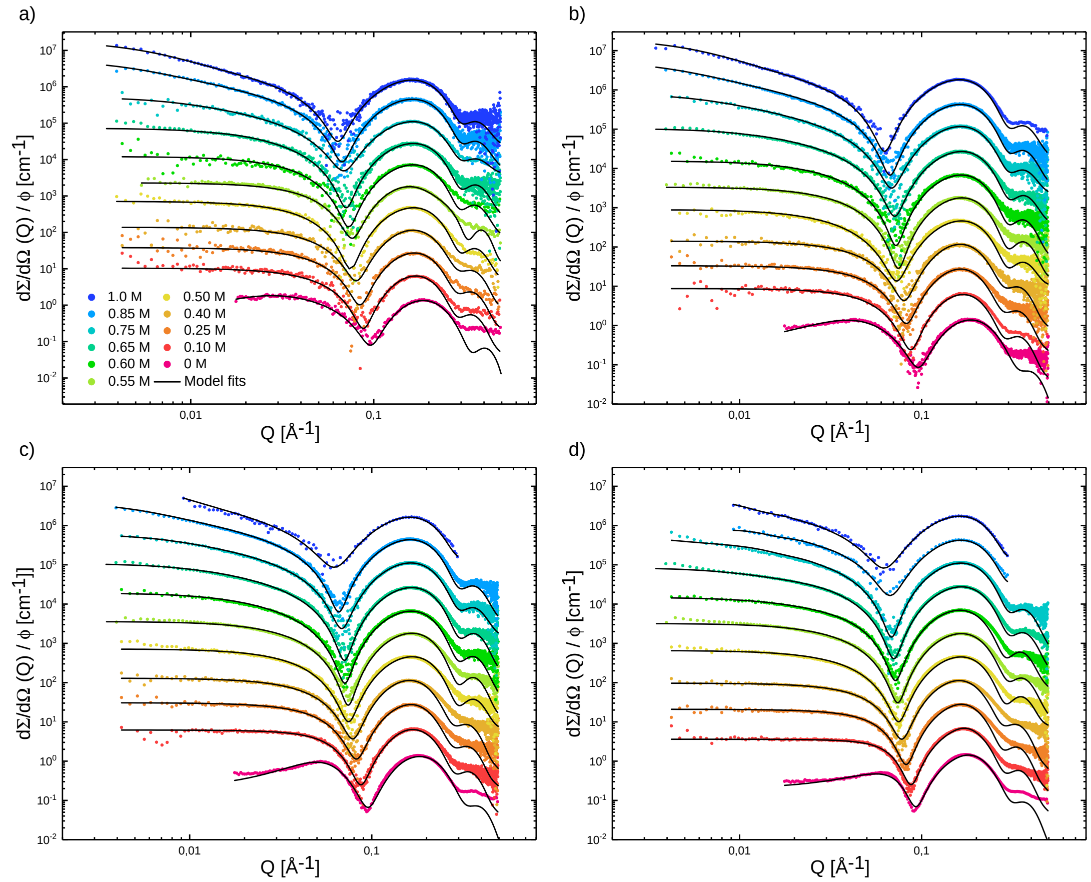

Figure S2: SAXS data for different concentrations of SDS at various amounts of salt (0 M to 1.0 M). For clarity, the data has been shifted vertically with factors 4^n where n goes from 0 to 10 from bottom to top. The solid lines display fits of the quantitative scattering models (see text for detail). All data is at 37 °C. **Panel a:** 22mM SDS. **Panel b:** 43 mM SDS. **Panel c:** 87 mM. **Panel d:** 173 mM SDS.

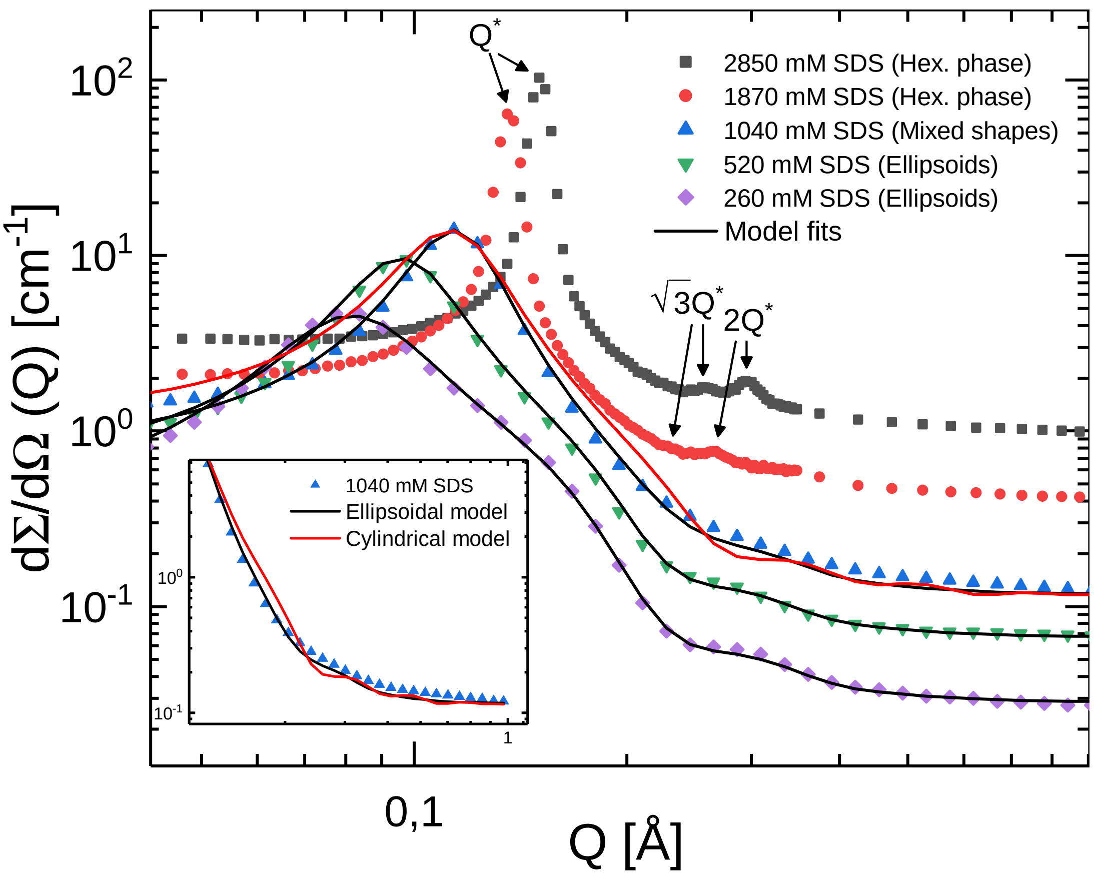

Figure S3: SANS data at higher concentrations of SDS in pure D_2O illustrating the transition from spheroidal micelles to cylinders packed in a hexagonal crystal phase (hcp). The solid lines display fits of the quantitative scattering models (see text for detail). The inset shows the magnified view of the scattering curve for 1040 mM SDS that cannot be readily described by the ellipsoidal or cylindrical models. This suggests a co-existence of SDS micelles of different morphologies and dispersities. All data is collected at 37 °C.

Fit parameters

Table S2: Fit parameters obtained from fitting the model of prolate ellipsoids with a Hayter-Penfold structure factor to SAXS curves from SDS in pure water. * denotes fixed parameters.

**Parameter**	**SDS in pure H_2O**			
Fit model	Ellipsoids			
$Conc.$ [mM]*	21.7	43.3	86.7	173
ϵ	1.49	1.45	1.39	1.36
R_{core} [Å]	12.4	12.4	12.8	13.6
t_{shell} [Å]	9.3	9.3	9.4	9.4
V_{head} [Å^3]*	60.1	60.1	60.1	60.1
R_{HS} [Å]	28.1	25.5	23.0	23.0
Z	8.2	8.0	12	12
η	0.005	0.019	0.029	0.075

Table S3: Fit parameters obtained from fitting the model of prolate ellipsoids with a Hayter-Penfold structure factor to SAXS curves from SDS in aqueous NaCl solutions (0.1 M, 0.25 M and 0.4 M). * denotes fixed parameters.

**Parameter**	**0.100 M NaCl**				**0.250 M NaCl**				**0.400 M NaCl**			
Fit model	Ellipsoid				Ellipsoid				Ellipsoid			
$Conc.$ [mM]*	21.7	43.4	86.7	173	21.7	43.4	86.7	173	21.7	43.4	86.7	173
ϵ	1.53	1.43	1.41	1.39	1.47	1.47	1.37	1.37	1.50	1.51	1.42	1.37
R_{core} [Å]	12.9	13.4	14.0	13.9	14.5	14.5	15.0	14.5	14.5	14.0	14.9	15.0
t_{shell}[Å]	9.09	9.60	9.70	9.70	9.40	9.40	9.30	9.42	8.90	9.65	9.50	9.60
V_{head} [Å^3]	63.0	63.0	62.3	63.0	64.4	64.4	64.4	64.4	65.1	65.5	65.5	65.5
R_{HS} [Å]	N/A	N/A	36.9	36.5	N/A	N/A	37.3	35.5	N/A	N/A	N/A	36.2
Z	N/A	N/A	0	0	N/A	N/A	0	0	N/A	N/A	N/A	0
η	N/A	N/A	0.06	0.121	N/A	N/A	0.02	0.06	N/A	N/A	N/A	0.04

Table S4: Fit parameters obtained from fitting cylinder models to scattering curves from SDS in aqueous solutions with varying concentrations of NaCl (0.5 M to 1.0 M). The PRISM structure factor was applied for all 173 mM SDS solutions. Note that certain values of $L_{c,mean}$ and b are not resolvable due to the limitations in the Q-range. Also, if $b > L_{c,mean}$, the micelles are considered to be worm-like. * denotes fixed parameters.

**Parameter**	**0.500 M NaCl**				**0.550 M NaCl**				**0.600 M NaCl**			
Fit model	Cylinder				Cylinder				Cylinder			
$Conc.$ [mM]*	21.7	43.4	86.7	173	21.7	43.4	86.7	173	21.7	43.4	86.7	173
R_{core} [Å]	13.1	13.1	13.1	13.8	12.8	12.8	13.0	13.1	13.1	13.0	13.0	13.4
t_{shell}[Å]	9.50	9.40	9.40	9.40	9.43	9.36	9.35	9.41	9.44	9.40	9.42	9.40
$L_{c,mean}$ [Å]	52.2	67.9	52.6	72.6	49.6	60.7	66.2	76.2	58.2	79.3	103	181
b [Å]	$> L_c$	$> L_c$	$> L_c$	$> L_c$	$> L_c$	$> L_c$	$> L_c$	$> L_c$	$> L_c$	$> L_c$	$> L_c$	$> L_c$
V_{head} [Å^3]	68.4	71.8	69.6	67.3	68.4	68.4	68.8	68.4	68.4	70.3	71.8	69.6
β	N/A	N/A	N/A	0.60	N/A	N/A	N/A	0.35	N/A	N/A	N/A	1.20
**Parameter**	**0.650 M NaCl**				**0.750 M NaCl**				**0.850 M NaCl**			
Fit model	Cylinder				Cylinder				Cylinder			
$Conc.$ [mM]*	21.7	43.4	86.7	173	21.7	43.4	86.7	173	21.7	43.4	86.7	173
R_{core} [Å]	13.0	12.8	12.8	12.8	12.8	12.8	12.8	12.7	12.8	12.8	12.7	12.4
t_{shell}[Å]	9.42	9.42	9.41	9.40	9.40	9.38	9.40	9.43	9.40	9.46	9.46	9.46
$L_{c,mean}$ [Å]	98.0	138	148	176	195	276	221	$> L_{max}$	502	726	$> L_{max}$	$> L_{max}$
b [Å]	$> L_c$	$> L_c$	$> L_c$	$> L_c$	$> L_c$	$> L_c$	$> L_c$	550	$> L_c$	$> L_c$	$> L_c$	600
V_{head} [Å^3]	71.0	71.4	71.8	71.9	73.0	73.0	73.0	71.8	74.5	75.6	75.6	75.6
β	N/A	N/A	N/A	0.50	N/A	N/A	N/A	8.00	N/A	N/A	N/A	4.10
**Parameter**	**1.00 M NaCl**											
Fit model	Cylinder											
$Conc.$ [mM]*	21.7	43.4	86.7	173								
R_{core} [Å]	12.0	12.8	12.2	12.6								
t_{shell}[Å]	9.67	9.61	9.60	9.63								
$L_{c,mean}$ [Å]	$> L_{max}$	$> L_{max}$	$> L_{max}$	$> L_{max}$								
b [Å]	434	462	498	592								
V_{head} [Å^3]	76.3	75.2	76.3	75.6								
β	N/A	N/A	N/A	1.95								

Table S5: Fit parameters obtained from fitting ellipsoidal and cylindrical models to SANS curves from SDS in pure D_2O. * denotes fixed parameters.

**Parameter**	**SDS in pure D_2O**			
Fit model	Ellipsoid			Cylinder
$Conc.$ [mM]*	260	520	1040	1040
ϵ	1.44	1.49	1.47	N/A
R_{core} [Å]	17.3	17.5	17.4	13.0
t_{shell} [Å]	8.4	8.4	8.4	8.4
$L_{c,mean}$ [Å]	N/A	N/A	N/A	65.0
V_{head} [Å^3]*	60.1	60.1	60.1	60.1
R_{HS} [Å]	25.6	26.0	25.8	N/A
Z	20	23	22	N/A
η	0.117	0.203	0.338	N/A
β	N/A	N/A	N/A	9.0

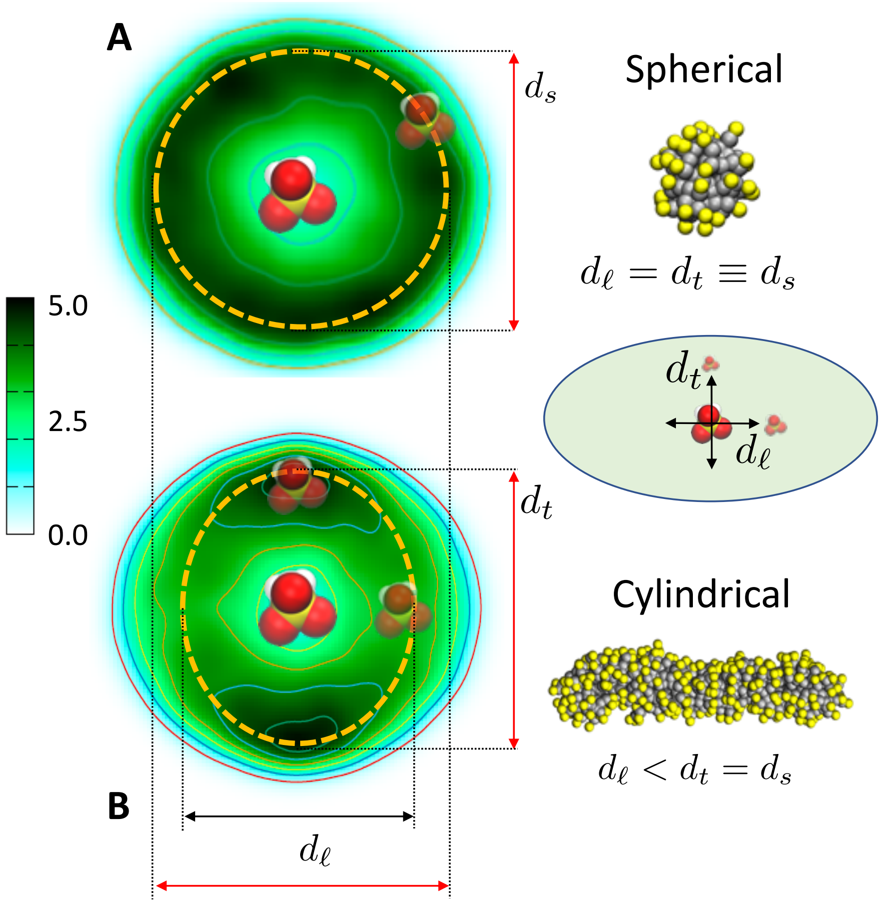

Figure S4: Density distribution of SDS heads around a reference one on the surface of a SDS micelle. A darker color corresponds to higher head density. In spherical aggregates, the density of first neighbors is symmetrically distributed in any direction at a distance d_s ≈8.6 Å. In cylindric ones, the first neighbors along the transverse direction are found at a distance $d_t \sim d_s$. On the contrary, the first neighbors along the longitudinal direction are found at a shorter distance d_ℓ (6.2 Å) smaller than d_s.

References

[55] H. C. Andersen, *J. Chem. Phys.* **1980**, *72*, 2384–2393.

[56] S. Vass, T. Torok, G. Jakli, E. Berecz, *J. Phys. Chem.* **1989**, *93*, 6553–6559.

[57] J.-F. Berret, *J. Chem. Phys.* **2005**, *123*, 164703.

[58] A. Guinier, Small-angle scattering of x-rays, eng, New York, **1955**.

[59] L. Rayleigh, *Proc. Royal Soc. London* **1910**, *84*, 25–46.

[60] J. B. Hayter, J. Penfold, *Mol. Phys.* **1981**, *42*, 109–118.

[61] J. Pedersen, M. Laso, P. Schurtenberger, *Phys. Rev. E* **1996**, *54*, R5917–R5920.

[62] J. S. Pedersen, P. Schurtenberger, *Macromolecules* **1996**, *29*, 7602–7612.

[63] L. Arleth, M. Bergström, J. S. Pedersen, *Langmuir* **2002**, *18*, 5343–5353.

[64] T. Neugebauer, *Ann. Phys.* **1943**, *434*, 509–533.

[65] P. Debye, *J. Phys. Colloid Chem.* **1947**, *51*, 18–32.

[66] M. E. Cates, S. J. Candau, *J. Phys. Condens. Matter* **1990**, *2*, 6869–6892.

[67] K. S. Schweizer, J. G. Curro in *Atomistic Modeling of Physical Properties*, (Eds.: L. Monnerie, U. W. Suter), Springer Berlin Heidelberg, Berlin, Heidelberg, **1994**, pp. 319–377.
